# Acetabular Insertion Avulsion Fracture of the Ligamentum Teres in a Child: A Case Report

**DOI:** 10.3390/diagnostics16203281

**Published:** 2026-10-09

**Authors:** Zhen-Zhen Dai, Taichun Li, Hai Li, Zi-Ming Zhang, Lin Sha

**Affiliations:** 1 Department of Pediatric Orthopedics, Xinhua Hospital, Shanghai Jiao Tong University School of Medicine, No. 1665 Kongjiang Road, Shanghai 200092, China; 2 Department of Orthopedics, Shanghai Children’s Hospital, Shanghai Jiao Tong University School of Medicine, No. 355 Luding Road, Shanghai 200062, China

**Keywords:** ligamentum teres, acetabular avulsion fracture, pediatric hip, hip arthroscopy, case report

## Abstract

**Background/Objectives:** Bony avulsion at the acetabular insertion of the ligamentum teres is rare in children and may be mistaken for a loose body. **Case Presentation:** A 12-year-old boy had persistent left hip pain and restricted motion after a road-traffic accident without documented dislocation. Radiographs suggested acetabular injury, prompting computed tomography (CT), which showed inferior acetabular irregularity and an intra-articular fragment. Magnetic resonance imaging (MRI), obtained to assess associated soft-tissue injury, showed effusion and periarticular edema but did not establish the fragment’s attachment. Arthroscopy identified a 15 × 10 mm notch-adjacent osseous fragment tethered to the ligamentum teres, with an intact femoral insertion and separate labral and chondral lesions. The fragment was excised because reliable arthroscopic fixation was not considered feasible under the operative conditions. After traction release, passive examination showed maintained concentric reduction without gross instability. At approximately 5 years, the modified Harris Hip Score was 100/100, pain was absent, and the patient had returned to sports; no contemporary imaging was available. **Discussion:** Arthroscopy distinguished a ligament-attached insertion avulsion from a free loose body. Previous excision reports provide a precedent, not comparative evidence favoring excision over fixation. **Conclusions:** Arthroscopy clarified the injury when imaging was indeterminate. The favorable outcome is limited to clinical and functional recovery, not long-term structural preservation.

## 1. Introduction

The ligamentum teres has a broad acetabular attachment spanning the notch and adjacent fossa, with fibers related to the transverse acetabular ligament [1,2]. Its specialized femoral insertion and neurovascular structures have also been characterized [3,4]; clinical reviews describe a secondary stabilizing role alongside the primary restraints provided by the capsule and osseous morphology [5,6]. Bony avulsion is distinct from the ligament tears and tendinopathy described in the broader literature [7]. Historical avulsion reports include injuries with and without documented dislocation [8,9,10], and recent pediatric reports provide relevant treatment and follow-up comparisons [11,12]. In children, an intra-articular osseous fragment may accompany a more extensive soft-tissue injury; computed tomography (CT) and magnetic resonance imaging (MRI) provide complementary information but may not establish its attachment [13,14].

This report complements previous pediatric cases by documenting a notch-side avulsion with preserved femoral attachment and a separate posteroinferior labral lesion, emphasizing the imaging–arthroscopy correlation and assessment of congruity and gross stability after excision.

## 2. Case Presentation

### 2.1. Patient Information and Clinical Findings

A previously healthy 12-year-old boy developed left hip pain and marked restriction of motion after a road-traffic accident. No hip dislocation or reduction maneuver was documented in the available emergency records or subsequent history. Pelvic radiographs obtained at a local hospital for the post-traumatic symptoms were interpreted as suggesting a left acetabular fracture. Conservative management was initiated, but persistent symptoms prompted referral for further evaluation.

Examination showed mild swelling and tenderness around the left hip, with intact skin and distal neurovascular function. Longitudinal percussion reproduced pain. Hip flexion was limited to approximately 90 degrees, abduction to 20 degrees, internal rotation to 20 degrees, and external rotation to 30 degrees.

### 2.2. Imaging Assessment

**Figure 1:**
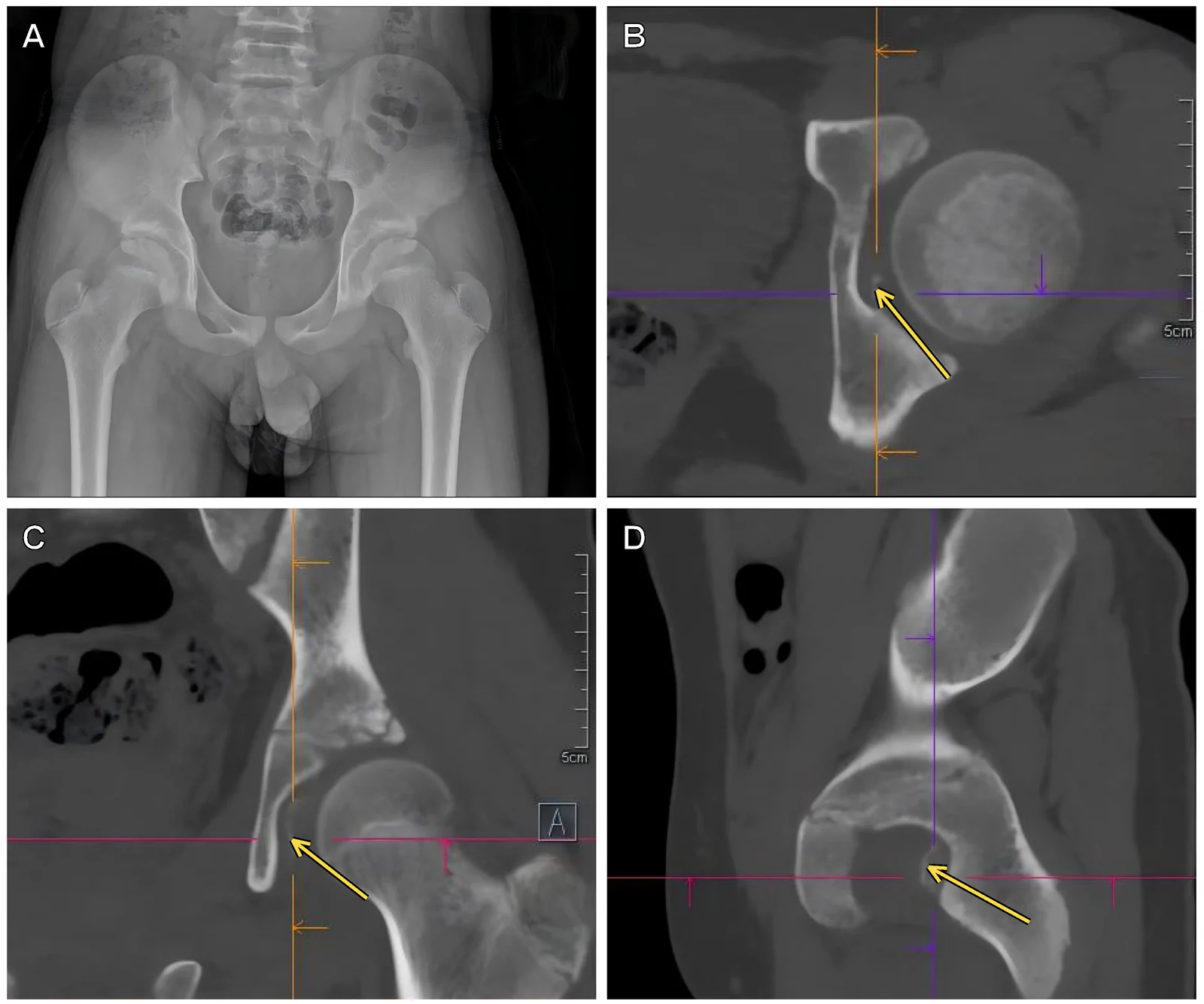
Preoperative radiography and CT. (**A**) Anteroposterior pelvic radiograph, initially interpreted as suggesting left acetabular injury; a discrete fracture line is not confidently resolved in this retained image. (**B**) Axial, (**C**) coronal, and (**D**) sagittal CT views cropped to the affected left hip. Yellow arrows in the CT panels indicate the intra-articular osseous density interpreted as the displaced fragment. Inferior acetabular irregularity also prompted further assessment; the exact donor site and ligamentous attachment were not established by CT. Existing colored cross-reference lines are scanner overlays, not lesion annotations.

**Figure 2:**
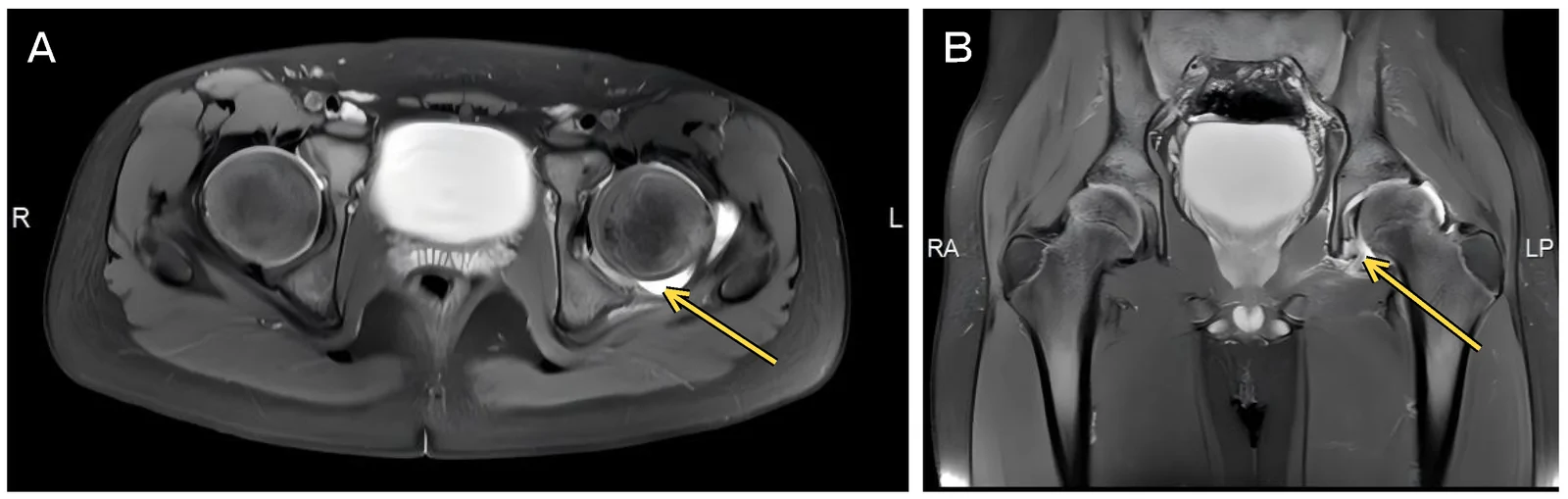
Preoperative MRI. (**A**) Axial and (**B**) coronal proton-density-weighted fat-suppressed images show left hip effusion (yellow arrows) and adjacent soft-tissue signal abnormality. The displayed images do not clearly demonstrate continuity between the fragment and the ligamentum teres.

The initial radiographs were interpreted as showing a possible left acetabular injury, although a discrete fracture line or donor site cannot be confidently localized on the retained radiographic image (Figure 1A). CT was obtained to characterize the suspected osseous injury and showed focal cortical irregularity of the inferior acetabular margin and a high-attenuation intra-articular fragment (Figure 1B–D). A displaced acetabular-margin fragment and a free intra-articular loose body were considered. MRI was used to evaluate associated intra-articular and periarticular soft-tissue injury; it demonstrated effusion and periarticular edema with preserved overall hip morphology (Figure 2). Neither modality established the fragment’s continuity with the ligamentum teres. Persistent pain, restricted motion, and an unresolved intra-articular lesion prompted diagnostic and therapeutic arthroscopy.

### 2.3. Arthroscopic Findings and Treatment

**Figure 3:**
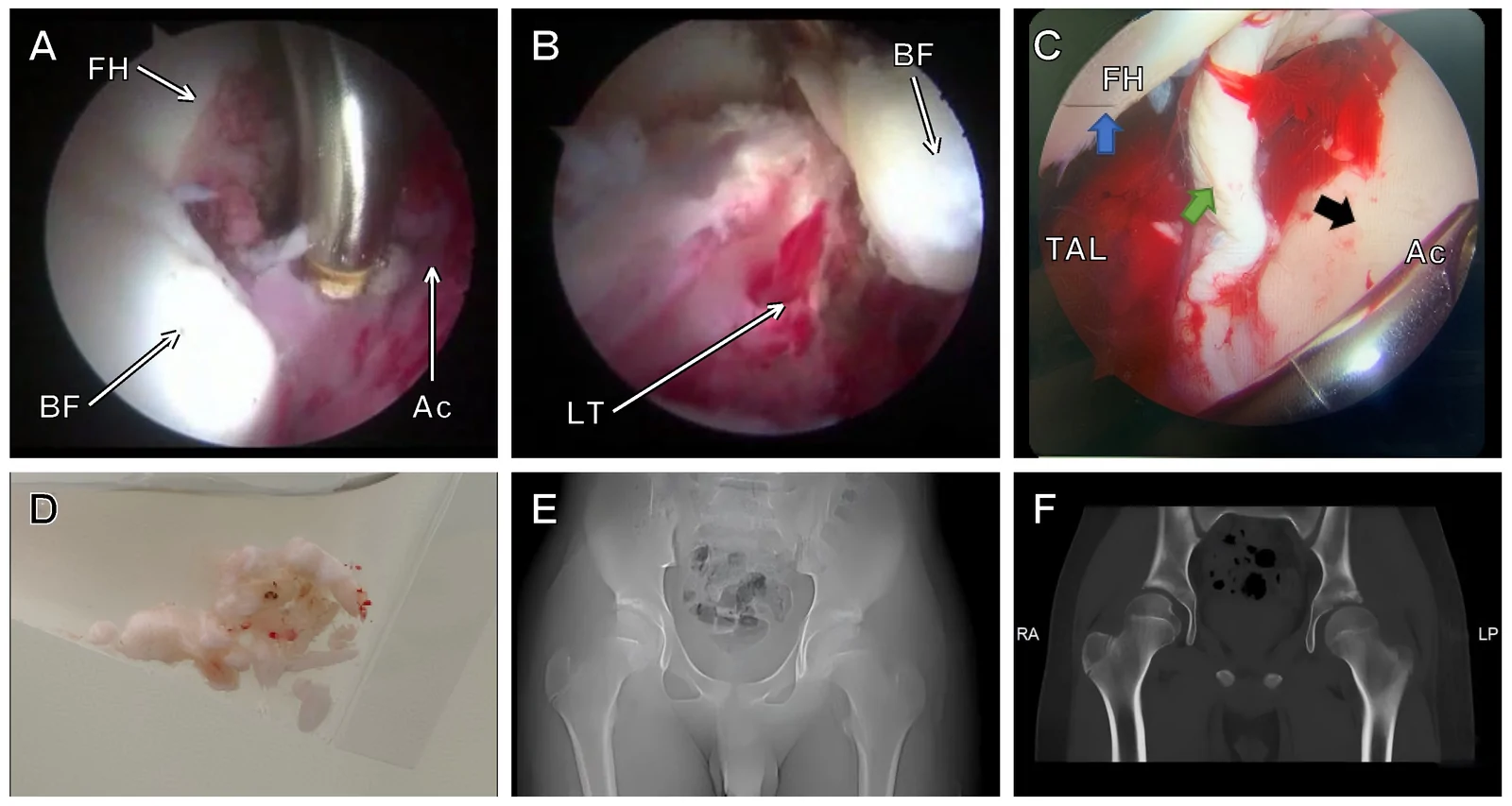
Arthroscopic findings and early postoperative imaging. (**A**) The avulsed bone fragment (BF) relative to the femoral head (FH) and acetabulum (Ac). (**B**) The fragment (BF) and ligamentum teres (LT) in the same arthroscopic field. (**C**) Acetabular-notch view; the original blue, black, and green arrows indicate FH, Ac, and the transverse acetabular ligament (TAL), respectively. (**D**) Excised tissue. (**E**) Anteroposterior CT localizer and (**F**) coronal CT image obtained on postoperative day 3. Alignment is congruent; no residual intra-articular osseous fragment is visible in the displayed coronal CT image. The stills do not fully delineate the donor site or complete ligament attachments; the separate posteroinferior labral lesion is not shown.

Arthroscopy was performed under traction through anterolateral, anterior, and posterolateral portals. Hemarthrosis, reactive synovitis, and synovial hyperemia were observed. After the field was cleared, a displaced 15 × 10 mm osseous fragment was identified in the central compartment, arising from the inferior acetabular margin adjacent to the notch. It remained attached to the acetabular side of the ligamentum teres, while the femoral attachment at the fovea capitis was intact. This established an acetabular-insertion avulsion fracture rather than a free loose body. A partial transverse acetabular ligament injury, a separate posteroinferior labral lesion, and multiple femoral-head chondral lesions were also documented. Representative arthroscopic views identify the fragment relative to the femoral head and acetabulum (Figure 3A), the fragment and ligamentum teres in the same field (Figure 3B), and the transverse acetabular ligament at the notch (Figure 3C). The exact donor site and complete ligament attachments are not fully delineated in these stills; the separate posteroinferior labral lesion is not shown.

The displaced, ligament-tethered fragment constituted a mechanical intra-articular lesion. Given the restricted working space around the acetabular notch and the treating team’s arthroscopic experience at the time, reliable arthroscopic reduction and fixation were not considered feasible. The fragment and attached avulsed tissue were therefore excised, with selective debridement of damaged tissue in the ligamentum teres–transverse ligament region (Figure 3D). The decision reflected the available arthroscopic conditions rather than demonstrated irreparability of the fragment.

No labral repair or cartilage-restoration procedure was performed. Formal grading and the detailed criteria for leaving these associated lesions unrepaired were not documented, precluding retrospective characterization of their stability or depth. After traction release, passive dynamic examination showed maintained concentric reduction without gross instability, supporting the decision not to add ligamentum teres reconstruction. The excised specimen was not examined histologically; the term “osseous avulsion fragment” reflects its CT and arthroscopic appearance.

### 2.4. Follow-Up and Outcomes

Radiography and CT on postoperative day 3 showed congruent hip alignment. No residual intra-articular osseous fragment was identified on the cross-sectional CT images; the CT localizer and a representative coronal CT image are shown in Figure 3E,F. Touch-down weight bearing with crutches was maintained for 4 weeks, followed by progressive rehabilitation. Pain had resolved and motion had improved at 1 week. At the 2-year clinical follow-up, the patient reported no pain or functional limitation, with clinically symmetric hip function.

At the latest follow-up, approximately 5 years after surgery when the patient was 18 years old, the clinical assessment was video-based. Pain on a visual analog scale (VAS) was 0 at rest and during activity. The modified Harris Hip Score (mHHS) [15] at this follow-up was 100/100, with full scores for pain (44/44) and function (47/47). The raw total of 91/91 was normalized to a 100-point scale as (raw total/91) × 100. He reported no hip or groin pain, limp, clicking, catching or locking, subjective instability, or apprehension. Daily activities and stair climbing were unrestricted, and he had returned to sports at his pre-injury level. Video assessment showed symmetric performance of deep squatting, single-leg stance, running, and jumping. No additional hip treatment or repeat surgery had been required, and he expressed satisfaction with the outcome. No contemporary radiographs or MRI were obtained, so late structural changes could not be evaluated. The clinical timeline is provided in Supplementary Table S1.

### 2.5. Case-Reporting Methods

This retrospective case report follows the CARE framework [16]; a checklist and clinical timeline are provided in the Supplementary Materials. Generative AI assisted with manuscript editing and literature review. The authors reviewed and take responsibility for the final content.

## 3. Discussion

The central diagnostic issue was whether the osseous fragment was free or remained attached to an injured intra-articular structure. CT localized the fragment near the inferior acetabulum, whereas MRI showed effusion and soft-tissue changes without resolving its attachment. Pediatric dislocation studies support MRI for associated unossified acetabular and soft-tissue injuries [13,14,17]; a systematic review also identified discrepancies between imaging and subsequent arthroscopic findings [18]. These studies inform interpretation but do not establish that a dislocation occurred in our patient. Here, arthroscopy resolved the attachment question and permitted treatment of the symptomatic lesion.

Table 1 summarizes selected directly relevant pediatric reports rather than an exhaustive review. Kashiwagi et al. and Matsushita et al. provide precedents for arthroscopic removal of an acetabular-sided, ligament-attached fragment [10,11]. Kolachala et al. described an incarcerated ligamentum teres avulsion with a separate posterior labral tear after documented dislocation, treated through surgical hip dislocation with fragment excision and labral repair [12]. Our case differs in its notch-adjacent origin, preserved femoral insertion, separate labral lesion, and recorded assessment of congruity and gross stability after excision. It does not introduce a new operative technique. No dislocation or reduction was documented, and the accident sequence cannot be reconstructed retrospectively.

The posterior acetabular “fleck” sign represents a different diagnostic problem. Blanchard et al. described a small osseous fleck associated with a larger posterior osteochondrolabral avulsion [19]; subsequent reports described repair of that complex [20,21]. In our patient, the notch-side fragment was attached to the ligamentum teres and separate from the posteroinferior labral lesion. Thus, neither small fragment size nor concentric alignment alone establishes the injured structure or the indication for repair. Evidence concerning posterior osteochondrolabral repair should not be treated as evidence about the same lesion reported here.

Pediatric arthroscopy reports describe individualized treatment of associated fragments and soft-tissue lesions but do not establish an excision threshold for this injury [22,23]. Excision in our patient reflected the restricted notch-side working space and arthroscopic expertise available at the time but did not demonstrate irreparability of the fragment. Fixation through another approach was not evaluated. Maintained concentric reduction after traction release supported omission of ligamentum teres reconstruction, not the preceding decision to excise. Passive examination cannot exclude microinstability. The reconstruction literature largely concerns selected patients with persistent symptomatic instability, often after previous surgery, rather than acute pediatric bony avulsion [24,25,26].

Associated labral and chondral injuries are relevant to both treatment and prognosis in pediatric hip trauma [27]. The separate lesions in this case were not formally graded, and detailed criteria for forgoing repair were not recorded. This prevents a reproducible comparison with the labral treatment reported by Kolachala et al. [12]. Neither their treatment nor our favorable result establishes that all such lesions should be repaired or left untreated; those decisions depend on lesion characteristics that are incompletely documented here.

The approximately 5-year mHHS of 100/100, absence of pain, return to sports, and symmetric video tasks support a favorable clinical and functional result. A maximum mHHS does not establish normal cartilage or fully characterize demanding sports performance. Validation of outcome instruments in pediatric and adolescent hip arthroscopy remains limited [28]; although our score was obtained at age 18, its applicability to this specific childhood injury is uncertain. Longer-term pediatric trauma reports reinforce the distinction between clinical function and structural sequelae [29,30]. Additional limitations are the retrospective single-patient design, absent histopathology, incomplete lesion characterization, and limited retained imaging. Video follow-up did not replace an in-person examination. Without contemporary radiographs or MRI, osteonecrosis, degeneration, remodeling, and growth-related abnormalities could not be assessed.

## 4. Conclusions

An osseous fragment near the acetabular notch may represent a ligamentum teres insertion avulsion rather than a free loose body, even without documented hip dislocation. In this symptomatic patient, arthroscopy clarified the fragment’s attachment and associated injuries. Excision was followed by maintained intraoperative congruity and a favorable approximately 5-year clinical and functional outcome. This result does not establish superiority over fixation or exclude late structural sequelae.

## Figures and Tables

**Table 1:** Selected pediatric reports of ligamentum teres avulsion compared with the present case.

Report/Patient	Diagnostic and Anatomic Features	Treatment	Reported Follow-Up
Kashiwagi et al., 2001 [10]; 10-year-old girl	Post-traumatic incongruity associated with interposed avulsed tissue; arthroscopy identified a ligament-attached acetabular-sided fragment.	Arthroscopic excision of the interposed fragment and avulsed tissue.	Asymptomatic at 1 year.
Matsushita et al., 2025 [11]; 8-year-old boy	CT showed an intra-articular fragment compromising congruity; arthroscopy identified an acetabular-fossa-side fragment attached to the ligament.	Arthroscopic removal of the ligamentum teres and bone fragments.	Favorable function at 14 years; remodeling and limb-length discrepancy were reported.
Kolachala et al., 2025 [12]; 13-year-old girl	Persistent symptoms after reduced posterior dislocation; CT identified an incarcerated fragment. Femoral-sided avulsion and a separate posterior labral tear were found.	Fragment excision through surgical hip dislocation and posterior labral repair.	Asymptomatic at 2 years.
Present case; 12-year-old boy	No documented dislocation. CT showed a fragment; MRI did not establish its attachment. Arthroscopy identified a notch-side avulsion with intact femoral attachment and a separate labral lesion.	Arthroscopic excision and selective debridement; no labral repair or ligament reconstruction.	Approximately 5 years: mHHS 100/100 and return to sports; no contemporary imaging.

CT, computed tomography; MRI, magnetic resonance imaging; mHHS, modified Harris Hip Score. This is a selected narrative comparison, not a systematic review or comparative treatment study.

## Data Availability

The de-identified data supporting this case are contained within this article. The video follow-up recording was reviewed for clinical assessment but was not submitted as Supplementary Material nor is it publicly available because it contains identifiable patient information.

## Supplementary Materials

The following supporting information can be downloaded at https://www.mdpi.com/article/10.3390/diagnostics16203281/s1, Table S1: Clinical timeline; Table S2: CARE checklist mapping to the manuscript.
