# Supplementary material for: Acetabular Insertion Avulsion Fracture of the Ligamentum Teres in a Child: A Case Report
Source: Diagnostics (Basel). 2026 Oct 9;16(20):3281. doi: 10.3390/diagnostics16203281 (PMC13703323; doi:10.3390/diagnostics16203281)
Supplement: Supplementary file 1 [file diagnostics-16-03281-s001.zip › diagnostics-4565395-supplementary.pdf]

Supplementary Material

Acetabular Insertion Avulsion Fracture of the Ligamentum Teres in a Child: A Case Report

Zhen-Zhen Dai; Taichun Li; Hai Li; Zi-Ming Zhang; Lin Sha

Table S1. Clinical timeline.

Time point	Clinical event or assessment
Injury	Road-traffic accident at age 12 years, followed by left hip pain and restricted motion. No hip dislocation or reduction maneuver was documented.
Initial assessment and referral	Local pelvic radiographs were interpreted as suggesting left acetabular injury. Conservative management was initiated; persistent symptoms prompted referral. The exact interval to referral and the conservative regimen and duration are not documented.
Preoperative assessment	CT demonstrated inferior acetabular cortical irregularity and an intra-articular osseous fragment. MRI showed effusion and periarticular edema. Neither established ligament–fragment continuity.
Index arthroscopy	A displaced 15 $\times$ 10-mm notch-side fragment remained attached to the ligamentum teres, with an intact femoral insertion. Partial transverse ligament injury, a separate posteroinferior labral lesion and femoral-head chondral lesions were observed. Restricted working space and the team's arthroscopic experience meant that reliable arthroscopic reduction and fixation were not considered feasible; excision and selective debridement were performed. No labral repair or cartilage-restoration procedure was undertaken.
After traction release	Passive dynamic assessment showed maintained concentric reduction without gross instability. Ligamentum teres reconstruction was not performed.
Postoperative day 3	Radiography and CT showed congruent hip alignment; no residual intra-articular osseous fragment was identified on cross-sectional CT.
Postoperative week 1	Pain had resolved and hip motion had improved.
First 4 postoperative weeks	Touch-down weight bearing with crutches, followed by progressive rehabilitation. Formal adherence assessment was not documented.
2-year clinical follow-up	No pain or functional limitation was reported; hip function was clinically symmetric.
Latest follow-up: approximately 5 years; age 18 years	Video assessment and reported outcomes: VAS 0 at rest and with activity; mHHS 100/100 (pain 44/44 and function 47/47; raw 91/91). No limp, mechanical symptoms or subjective instability; unrestricted daily activity and return to pre-injury sports. Deep squat, single-leg stance, running and jumping appeared symmetric. No additional hip treatment or repeat surgery was reported. No contemporary radiographs or MRI were obtained.

Timing follows the relative intervals in the clinical record; exact calendar dates are not supplied. CT, computed tomography; MRI, magnetic resonance imaging; VAS, visual analog scale; mHHS, modified Harris Hip Score. The mHHS raw maximum is 91; the normalized score is (raw total/91) $\times$ 100.

Table S2. CARE checklist mapping to the manuscript.

CARE 2013 items are summarized below. Status reflects the available report and records, including incomplete information. Locations refer to the manuscript; Section 3 (Discussion) has no subsections.

Item	Topic	Status	Manuscript location and reporting note
1	Title	Reported	Title identifies the injury and includes "A Case Report."
2	Keywords	Reported	Five keywords, including "case report."
3a	Abstract: contribution	Reported	Background/Objectives and Discussion: diagnostic distinction between a loose body and insertion avulsion.
3b	Abstract: presentation	Reported	Case Presentation: post-traumatic pain, restricted motion and key imaging and arthroscopic findings.
3c	Abstract: management and outcome	Reported	Case Presentation: fragment excision, intraoperative stability and approximately 5-year clinical results.
3d	Abstract: lesson	Reported	Discussion and Conclusions: arthroscopic identification and restriction to clinical and functional recovery.
4	Background	Reported	Section 1: anatomy, rarity, prior reports and contribution of this case.
5a	Patient details	Reported	Section 2.1: age, sex, affected side and injury context, without direct identifiers.
5b	Symptoms	Reported	Section 2.1: persistent hip pain and substantial restriction of motion.
5c	Relevant history	Partial	Section 2.1: previously healthy. Detailed family, genetic and psychosocial histories are not available in the retained records.
5d	Earlier treatment	Partial	Section 2.1: initial conservative management and persistent symptoms. The specific regimen and duration are not documented.
6	Examination	Reported	Section 2.1: tenderness, swelling, distal neurovascular findings and recorded motion limits.
7	Chronology	Reported	Table S1: injury, assessment, intervention and follow-up using documented relative intervals.
8a	Diagnostic methods	Reported	Sections 2.1–2.3; Figures 1–3: examination, radiography, CT, MRI and arthroscopy.
8b	Diagnostic uncertainty	Reported	Section 2.2 and Discussion: imaging did not establish the fragment's precise attachment.
8c	Diagnostic reasoning	Reported	Sections 2.2–2.3 and Discussion: acetabular-margin fragment/free loose body considered; arthroscopy identified insertion avulsion.
8d	Prognostic descriptors	Not applicable	No formal prognostic staging is applied to this traumatic avulsion case.

Checklist source: CARE Case Report Guidelines, 2013 CARE Checklist. Item descriptions are condensed; this table is a manuscript-specific reporting map.

Table S2. CARE checklist mapping (continued).

Item	Topic	Status	Manuscript location and reporting note
9a	Intervention type	Reported	Section 2.3 and Figure 3: arthroscopic fragment excision and selective debridement; no ligament reconstruction, labral repair or cartilage-restoration procedure.
9b	Intervention details	Partial	Sections 2.3–2.4: portals, traction, operative steps and 4-week protected weight bearing. Initial conservative treatment details and formal grading of the associated lesions are unavailable.
9c	Treatment changes and reasons	Partial	Sections 2.1–2.3: persistent symptoms prompted arthroscopy; technical constraints led to excision. Detailed criteria for forgoing labral repair and cartilage restoration are not documented. No additional hip treatment was reported.
10a	Clinical and patient-reported results	Reported	Section 2.4: early improvement, 2-year clinical findings and approximately 5-year video assessment; VAS 0 and mHHS 100/100, with symptoms and activity reported.
10b	Follow-up investigations	Partial	Section 2.4: day-3 radiography and CT; Figure 3E,F shows the CT localizer and a coronal CT image. No contemporary imaging at the latest follow-up; late structural changes cannot be assessed.
10c	Adherence and tolerability	Not formally assessed	Section 2.4 reports protected weight bearing and rehabilitation. A formal method for assessing adherence or tolerability is not documented.
10d	Adverse events	Partial	Section 2.4: no additional hip treatment or repeat surgery reported. Absence of late structural complications cannot be established without current imaging.
11a	Strengths and limitations	Reported	Discussion: direct arthroscopic diagnosis; retrospective single case, limited retained documentation, ungraded associated lesions, absent histology and latest imaging.
11b	Literature context	Reported	Discussion and Table 1: selected avulsion reports, pediatric imaging, alternative injury patterns, and treatment evidence.
11c	Basis for interpretation	Reported	Discussion: diagnostic attachment, patient-specific treatment constraints, post-excision congruity and limits of causal inference. An undocumented transient dislocation cannot be confirmed or excluded.
11d	Main lesson	Reported	Section 4 (Conclusions): insertion avulsion may mimic a loose body; arthroscopy clarified this case, while the outcome does not establish superiority of excision or structural preservation.
12	Patient's perspective	Partial	Section 2.4 records satisfaction as conveyed by the authors. A patient-authored account of the experience is not available.
13	Publication consent	Reported	Informed Consent Statement: written consent for publication of the case and accompanying clinical images was obtained.

Figure mapping in the manuscript: Figure 1, radiograph and CT; Figure 2, MRI; Figure 3A–C, arthroscopic findings; Figure 3D, excised tissue; Figure 3E, anteroposterior CT localizer; Figure 3F, coronal CT image, both obtained on postoperative day 3. Main-text Table 1 is the selected case comparison; the clinical timeline remains Table S1. The CARE therapeutic-intervention domain contains items 9a–9c.
